# Winchester County Hospital
*Prov. Med. Gaz. No. 11.


**Published:** 1830-01-01

**Authors:** 


### WINCHESTER COUNTY HOSPITAL.*

#### I. VIPER BITE—SEVERE LOCAL AND CONSTITUTIONAL SYMPTOMS — EMPLOYMENT OF CUPPING-GLASSES.

John Pound, ten years of age, was brought to the hospital, on the evening of the 24th of June, with general ecchymosis and tension of the whole foot, leg, and inferior part of the left thigh, the integuments of which were polished and transparent, but not œdematous. On minute inspection five distinct dental punctures were readily discerned contiguous to each other, somewhat elevated, and surrounded by an inflammatory blush—the countenance was anxious—tongue coated with a dense brown fur—pulse small, irregular, and slightly intermittent—temperature of the limb somewhat reduced. At nine o'clock that morning he had been bitten in the leg by a viper in a plantation, immediately after which there succeeded a rapid and extensive swelling of the foot and leg, extreme burning pain and tension. He had suffered much from acute pain in the epigastrium, with retching and vomiting of frothy mucus.

Mr. Henry Lyford, under whose care the patient was placed, directed a cupping-glass to be applied immediately over the punctures. A few drops of serous fluid exuded, and three small vesications appeared, when the scarificators were had recourse to, and a small quantity of blood abstracted with little or no good effect. The leg was fomented during the night, and a draught of spt. ammon. aromat. and tincture of opium in camphor mixture directed to be taken every five hours during the night, with house physic in the morning. He obtained no rest, and on the 25th the tension had extended up the thigh—the temperature of the body and limb was increased—the pulse 113, and fluttering—the tongue coated with a brown fur in its centre—partially dry, and red at the edges. The aperient not having acted, some castor oil was given, but at 8, p.m. this also had failed to move the bowels. The ecchymosis and tension had now encroached on the abdomen and perineum to such an extent, that the scrotum was quite obscured—a number of minute vesications had made their appearance below the knee—the heat of skin and thirst were aggravated, but the state of the tongue and pulse was improved. Spirit lotion was applied to the legs, and salts were prescribed every three hours till the bowels should be opened. By 8, a.m. of the 26th, the medicine had acted twice copiously. The ecchymosis, &c. had now reached the left lumbar region and commenced invading the right side of the abdomen, which was tender to the touch. The linimentum camphoræ was substituted for the spirit lotion, and the aperient was continued. From this time the unfavourable symptoms gradually subsided, and on the 1st of July the whole extremity, though discoloured, was reduced to its natural size, and the patient made no complaint but of extreme weakness and stiffness of the leg. The following remarks appended to the case appear to be so just, that we are tempted to transcribe them entire.

"The train of symptoms which manifested themselves throughout the progress of this case, might be clearly referred to two causes; namely, the injury inflicted on some of the branches of the deep-seated absorbents, by the fangs of the viper producing, as a consequence, the local effects, inflammation of these vessels, which rapidly extended to the larger trunks accompanying the great vessels; and, secondly, the action of the poison injected into the wound producing its morbid and specific operation on the constitution, through the medium of the nervous system—the vast accumulation of serous fluid, indicated by the tension which existed from the commencement to the termination of the complaint, depending, no doubt, on a diminished and impaired action of the absorbents—the acute pain produced, exclusively, by pressure made throughout their course up the limb—the evident benefit derived by the stimulating effects of the

---

* Prov. Med. Gaz. No. 11.

camphor liniment on the skin—all coincide in confirming the supposition entertained respecting the pathological condition of the affected parts. Accidents of this description are certainly unfrequent within this district: some few cases, however, have occurred, the great proportion having been attended with precisely similar results as in the foregoing narrative; but when the superficial absorbents have been the more immediate seat of injury, an erysipelatous inflammation has quickly supervened throughout their course—an example of which we not long since had an opportunity of witnessing in the person of a noble earl, of robust stature, who was bitten in the fore-arm. A high degree of erysipelas almost immediately ensued, which, in the course of a few hours, extended into the axilla, producing severe constitutional irritation. These unpleasant symptoms, however, in three or four days, began gradually to decline, and, at the expiration of a week, had entirely disappeared, leaving the patient convalescent.

"We feel no hesitation in acknowledging our incredulity and scepticism respecting the frequent fatality so very commonly supposed to be the result of the bite of these reptiles, in this country at least; and, indeed, our recollection fails at present in furnishing us with any well-authenticated instance of death from this cause. We do not, however, for an instant pretend to deny the possibility of such an event, but simply to state our conviction, that when death does ensue, it is the consequence of a peculiar irritability of constitution, rather than from the activity of the viperine poison. The climate of Great Britain is well known not to be, by any means congenial to the full and complete development or perfection of the reptile species: if, therefore, it be admitted—and there appears no reasonable ground for dissent—that the virulence and intensity of the deleterious secretions of these creatures are intimately dependent on, and modified by, the vigour of their constitutional powers, but little difficulty can remain, by way of satisfactory explanation, as to the comparative inertness of the poison of the viper, indigenous to this country, when contrasted with the deadly venom of the same tribes, in more remote but less favoured regions. It has been ascertained, by actual experiment, that dogs may swallow with impunity much larger quantities of the poisonous secretions than Fontana supposed, and that, if it be kept for some length of time, it loses its accustomed acrimony.

"We subjoin the following condensed account for the information of those who may not have devoted their attention to the natural history of the viper:

"*Vipera communis*, common viper—*coluber berus* of Linnæus. Characteristics: Maxillary bones, with poison fangs, but no common teeth—scales behind the vent divided—neck narrow—head destitute of plates—dorsal scales, oval, carinated—inferior lateral ones subangular and plain—length from two to three feet—colour, dirty yellow—a stripe on each side, of black triangular spots, and a dorsal stripe of confluent, rhomboidal spots—space between the eyes, and two spots on the crown, black—the head is broad behind—edges of the jaws covered with large scales—belly dusky, tinged with blue—scales on the belly, 142 to 148—pairs on the tail, 30 to 40—oviparous, producing from 12 to 25 young—feeds on insects, frogs, and mice—becomes torpid during the winter. It should be recollected, however, that the markings on its body depend, in some degree, upon age, sex, and season."

### II. Cataracts alternating with Diabetes.

The following is a very singular case, and one that would be met with incredulity, were it not guaranteed as it is, by the name of the surgeon and of the hospital.

Eliza Broomfield, æt. 15, remarkably tall and thin, was admitted with cataract in either eye. The pupils were dilated to their utmost, and the crystalline lens was evidently so augmented in bulk as to protrude through the pupillary opening; its colour was uniformly milky; the cornea unusually glassy; the vision so completely lost that the patient could only distinguish most imperfectly light from darkness. Independent of the cataracts, there were dyspnœa, cough, and loss of appetite.

The cataracts had appeared simultaneously fourteen months previous to her admission, and had been completely formed in the surprisingly short period of twenty days. The menses had appeared at the age of eleven, and had flowed with regularity till her thirteenth year, when they disappeared. From having been healthy and robust, she now became very debilitated, increased most astonishingly in stature, and subsequently was attacked with profuse nocturnal perspirations. When twelve months had elapsed an evident amendment took place, but it proved to be temporary only, and very shortly afterwards the girl began to complain of uneasiness about the head, with vertigo, confusion, and obscurity of vision.

℞. *Infus. gent. c.* ℥j. *Spt. æth. nit.* ʒss. *Tr. camph. c.* ʒss. *ter die sumend. Pil. hyd. gr.* ij. *omni nocte sumend.*

These medicines were continued for upwards of a fortnight with considerable benefit, and on the 20th of February we find that, though the cough was nearly gone, the dyspnœa and appetite were worse. The dilatation of the pupils, bulging, and milky whiteness of each lens were diminished, and the patient could readily detect any substance interposed between her and the light from the window. The medicines were repeated with the addition of an aperient. On the 24th she could discern the flame of a candle at some little distance, but it was now discovered that the urine was too abundant.

"*Feb. 28th.*—The quantity of urine passed for the last four days has averaged sixteen pints daily. Its colour of a greenish yellow, and its taste strongly mellitic. Pulse 65, and feeble; appetite natural; dyspnœa not diminished; the emaciation very considerable; tongue morbidly clean; skin dry and scurfy. The cataracts are rapidly disappearing. Ordered to subsist exclusively on animal food, and to substitute the following medicines for those which the patient has been taking:

"℞. Sulph. zinci, ʒss.

Extract. cinchonæ, ʒiss. ft. pil. xxiv. sumat ij. ter die c. haust. sequent.

"℞. Tinct. opii, gtt. vj.

Decoct. cinchon. ℥j.

Conf. aromat. ℈j. ft. haustus.

"*March 3d.*—Urine passed the two last days, thirteen pints and a half—pulse 72—the cataracts have totally disappeared, and the patient's visual powers perfect; being enabled to employ her needle with perfect ease.—Continue medicines and diet.

"*March 9th.*—Urine has decreased to nine pints—pulse 94—the dyspnœa has very much abated, and there is a decided amendment in the patient's strength. Continue.

"*March 14th.*—Urine, eight pints—pulse 88.

"*18th.*—Urine, 7 pints—pulse 96.

"*25th.*—Urine, two pints, and natural in respect to colour and taste—pulse 80. The sole complaint appears to be extreme debility. The patient having regained her sight, and having been relieved of her diabetic symptoms, she became anxious to return home to her friends. She was accordingly discharged, with an injunction to come back to the Hospital, should any of her former symptoms recur.

"*May 10th.*—Intense interest, as might naturally be expected, was excited with respect to the probable issue of this extraordinary case, which did not in the least subside after the departure of the patient from the house. Frequent opportunity was, fortunately, afforded of enquiry concerning her from a relative who resided in an adjoining house, and who visited the Hospital once a fortnight as an out-patient. From this person, the following report was obtained, and has, subsequently been confirmed by the testimony of the mother of the patient.

"From her narrative, it appears, that almost immediately (about four days) after the young woman had returned home, she began to experience a relapse of uneasiness about the head, accompanied with great obscurity of vision, which increased with such rapidity, that, before a week had elapsed, she was in a state of utter darkness.

To this pitiable condition succeeded her diabetic symptoms, but in a more aggravated form than in her previous attack, together with rapid emaciation, and overpowering debility. The quantity of urine evacuated for several days prior to her decease averaged at twenty pints per diem; but, with the accession of these symptoms, there was a complete restoration of the natural functions of the eye, which she retained most perfectly to the last moments of her existence. Her death took place four weeks after the return of the diabetes. There had been no post-mortem examination."

III. Dislocation of the Clavicle forwards. Reduction at the Expiration of Twelve Weeks.

James Savage, fifty years of age, fell with his left arm outstretched in such a manner that it bore the whole brunt of the violence. Inability to raise the arm as before, and "an *intense dull swelling*, which after a time became almost imperceptible from the enormous degree of tumefaction which ensued," were the consequences of the accident. No attempt at reduction was made, and twelve weeks after the occurrence of the accident he entered the Winchester Hospital under the care of Mr. Lyford. On the superior part of the sternum was a distinct obtuse projection, exquisitely sensible when touched, and attended with slight inflammation of the integuments; this projection could readily be traced as a continuation and termination of the clavicle, though the motions of the shoulder appeared to produce no alteration in its situation. The motions in question were painful; the shoulder itself had a decided inclination forwards, and the distance between its point and the mesial line of the sternum, was shorter, on admeasurement, than in the opposite extremity.

"The treatment consisted in the application of the clavicle bandage with pads under the axilla. The shoulders were drawn backwards, as far as they could be, which was not, however, to the fullest extent, the patient having acquired, from his agricultural pursuits, a somewhat prominent back, so that the bases of the scapulæ were farther asunder than natural. The effect of the bandage was not that of restoring the dislocated extremity of the clavicle immediately into its proper receptacle on the sternum. This object was accomplished, in the most gradual manner, by tightening the bandage every four or five days, until the scapulæ were completely approximated. The patient was confined in bed for three weeks on his back, which greatly assisted the bandage in its office of retaining the shoulders in the wished-for position. Moderate pressure was made by the application of soap-plaister on the dislocated parts; and, at the expiration of a month, the parts had acquired their original and natural situations."

IV. Dislocation of the Head of the Radius backwards.

It is well known that Sir Astley Cooper in his magnificent work on Dislocations has ranked dislocation of the head of the radius backwards as extremely rare. Sir Astley, indeed, if we remember right, never witnessed more than one or at most two cases of the accident in question. Since the publication of the work, however, we have reason to know that an instance or two of the kind has been shewn to the worthy Baronet, and several have been noticed at different times in this Journal. Mr. Case now communicates two further cases, which, being very brief, we shall give to our readers in the reporter's own words. The many examples of this dislocation now upon record are sufficient to shew that it is much more frequent than Sir Astley Cooper has imagined, and prove how incomplete is the greatest experience that any individual can hope to attain. It is by collecting and collating stray facts of this description that periodical literature may prove of much service to professional men and to science.

"*Case* 1. William Smith, ætat. 14, was admitted in the month of November, 1828, on account of a disease of the tibia. On examination of his right elbow, a dislocation of the radius from the ulna was discovered, which, it appeared, had been treated for an abscess, there being, on each side of the head of the bone, a cicatrix of some extent. The injury took place when the patient was an infant; he, therefore, can give no account of it. The appearances which presented themselves were as follows: The elbow was very much deformed, the head of the radius being thrown upon the external condyle of the humerus, making a large projection there; he is unable to make perfect flexion or extension of the fore-arm upon the humerus; neither is it in his power to perform the actions of pronation and supination, as there is not the least rotatory motion of the radius; and, when requested to turn his hand upwards and downwards, the whole motion is performed at the shoulder-joint."

"*Case* 2. John Hewsted, ætat. 21, was admitted on the first of April, 1829, with an eruption, extending over the greater part of his body. On exposing his left arm, great deformity of the elbow-joint was observed. He gives the following account of its origin: About thirteen years ago, whilst playing at cricket, he struck the end of the bat against the ground, which accident caused immediately the present unshapely appearance of the joint. Nothing was done for him at this time, the surgeon, to whom he applied, not being acquainted with the nature of the injury.

"At the time of the accident, he was able to bend the arm in a slight degree, but that motion has now ceased.

"In the year 1826, an abscess formed about the joint, which was opened. Blisters were applied at the same time, but without any relief.

"On examination, it proved to be a dislocation of the radius, rather laterally and backwards, the head being thrown upon the humerus between its articulating surface, and the extreme point of the external condyle. Perfect anchylosis of the joint has taken place, leaving the arm in the semiflexed position, without the least degree of motion in any direction whatever."

### V. Renal Calculi—Laryngitis—Abscess external to the Larynx.

The following case is deserving of consideration on account of the fatal suppuration external to the larynx, a circumstance which we know to have occurred in several instances. Not very long ago it took place in a private patient of the Editor of this Journal, and excited considerable interest and attention. At some future opportunity we may make some remarks on this curious affection, but at present we have not the leisure to do more than hint at its comparative frequency.

The subject of the case upon the *tapis*, was a labourer, ætat. 62, admitted with fixed, obtuse pain in the right lumbar region, occasionally aggravated into intolerable paroxysms—micturition was frequent. He attributed his complaint to a severe kick from a horse received in the flank some months previously—he had occasionally voided some blood in his urine, but never any sand or gravel. Cupping, the warm-bath, and other appropriate means were employed, when at the expiration of a fortnight, he exposed himself to a current of air from an open window, and was seized with what seemed to be a cold. Five grains of calomel were given, apparently with benefit, but he soon complained that his throat was very sore, and on inspecting the fauces, the uvula and tonsils were found to be slightly inflamed. There were no febrile symptoms, and he was merely ordered a gargle of tincture of myrrh in infusion of roses. Next morning, however, a fatal change had occurred;—the respiration was quick, laborious and impeded, with total inability to swallow or speak—the pulse was rapid and feeble—the countenance flushed and intensely anxious—the recumbent posture impossible. There was an evident external fulness and tenderness on pressure about the throat, and on examining it internally the inflammation was found to have deserted the uvula and tonsils for the epiglottis, which was seen to be highly inflamed, swollen, and altered in form; the back part of the mouth seemed also clogged with a thick mucus. The patient was now transferred to the physician to treat the laryngitis set up, which was done by the most active antiphlogistic treatment, but without avail, for in less than forty hours from the commencement of the attack the patient died. The medicines were necessarily exhibited by means of the stomach-pump.

"*Sectio Cadaveris.* The body was inspected about ten hours after death, when the tongue, larynx, and pharynx were removed, in order that they might undergo a more accurate and special examination. The epiglottis had become prodigiously augmented in size, from serous effusion between its substance and the membrane by which it is invested; it had become likewise perfectly inelastic, convex on either side, so that its natural adaptation to the glottis was completely destroyed. The larynx and trachea on their inner surface presented evident traces of inflammation—the whole membrane being covered by florid vessels, giving a general and regular appearance of increased vascularity; but the effusion of coagulable lymph, so frequently succeeding inflammation of these parts, had not occurred. On separating the larynx from the pharynx, a large abscess was detected, containing about an ounce of healthy pus—the walls of the abscess being formed by the cellular tissue interposed between these tubes. The examination was now directed towards the lumbar region, with the view of ascertaining the exact cause of the disease for which he had been admitted into the hospital. The abdominal cavity was, therefore, opened, and all the viscera, with the exception of the kidnies, found to be healthy. The right kidney contained a singular shaped calculus, the size of a walnut; it had partly emerged from the pelvis of the kidney into the commencement of the urethra, which had become prodigiously dilated. The left-kidney contained a number of minute calculi; the largest not exceeding in diameter the size of a pea. The structure of each kidney was more than usually firm, and highly vascular. The ureter on the right side was more or less covered with tortuous distended veins.

"The thoracic viscera healthy—head not examined—the right kidney, with its calculus, has been preserved and placed in the hospital museum."

### XXXIX.

### WINCHESTER COUNTY HOSPITAL.

### FUNGUS HÆMATODES.*

The diagnosis of this horrible disease is frequently so obscure that it is an object to place before the public as many well authenticated cases as possible. To those who are connected with great hospitals the facts thus stored up are not perhaps of vital importance, as they see not a few of the same kind in their pauper practice. But to those who have not such opportunities for seeing cases, the power of reading them becomes a matter of great consequence, and to that class of readers we would especially address ourselves. Persons will sometimes ask with great simplicity or would-be wit, the cui-bono of publishing cases of malignant disease,

* Prov. Med. Gaz. No. II.

and inquire how such information helps them towards the cure. If such querists were at all acquainted with practice, they would know that an accurate diagnosis not seldom obtains a man more credit than the most scientific and masterly treatment. Of the truth of the former every one can judge, but any ass may cavil at the latter. With these remarks we proceed to the consideration of some interesting cases of fungus hæmatodes, recorded in our esteemed Provincial contemporary.

CASE 1. *Fungoid Tumour of the Eye—return of the Disease after extirpation.*

G. Merrel, æt. 40, labourer, admitted with the globe of the left eye enlarged to three times its natural size, projecting from the orbit, uncovered for a space of three inches by the palpebræ, which are pressed backwards against the arch of the orbit and form a complete girt round the base of the diseased mass. The palpebræ are œdematous and livid in colour; the globe has entirely lost its natural structure, being of fleshy colour, medullary structure, and mottled on its surface with varicose veins. In the centre of the diseased globe is a deep black slough from which a bloody sanies constantly exudes and excoriates the integuments of the cheek over which it passes. He has occasional deep and lancinating pain extending to the temple and ear of the left side, and occasionally "into the substance of the brain itself." Considerable emaciation, anxiety, and dejection of countenance—sallow lemon-coloured complexion — sleep broken with convulsive twitchings — appetite impaired — pulse 96 — bowels open. There is no glandular enlargement.

He dates the commencement of his complaint to a severe blow on the eye from a cricket ball received six months ago. An acute attack of inflammation succeeded the injury, and five weeks afterwards a second attack followed which ended in the present disease. The nature of his case being fairly explained to him, and the slight chance of success which was promised even from the operation, the patient determined to try what the knife would do. Accordingly in ten or twelve days from his admission extirpation of the eye was performed in the following manner.

"The patient being laid in the recumbent posture on his back, with the head slightly elevated, the operator placed himself behind, and commenced by making a transverse incision at the external canthus, so as to extend the fissures of the palpebræ, and thereby to afford, by giving more space, a greater facility for the extirpation of the diseased parts; a curved needle, armed with a thick ligature, was then introduced through the centre of the swelling, and the ends held firm by an assistant. A convex double-edged scalpel was next inserted deep into the orbit, immediately below the orbitar process of the os frontis, and carried around the circumference of the diseased parts, including the lachrymal gland; this incision being completed, the eye was drawn forwards by the assistant, which exposed the optic nerve, which was divided, and the extirpation concluded. The operation was no less difficult than unsatisfactory, from the firm, deep-seated, and extensive adhesion, and communication of the diseased parts, with the periosteum lining the vault of the orbit. No hæmorrhage of consequence ensued. The palpebræ were approximated — simple dressing, with a compress wetted in zinc lotion, applied—the patient removed to his bed, and an anodyne draught exhibited."

The patient went on exceedingly well, was put upon the bark, and in fact became in every respect convalescent. Unfortunately, however, these cheering prospects were blasted and the patient's hopes darkened by a return of the lancinating pain in the orbit, in the night of the 39th day after the operation. The spirits now became depressed, the strength broke down, and hectic fever was established. The upper palpebra swelled, the lancinating pains grew more severe, fungous granulations sprouted from the orbit and occasioned more disfigurement than the original disease, and on the 54th day the poor fellow left the hospital at his own request in a pitiable state.

The section of the tumour, as frequently happens, gave no satisfactory information respecting the origin of the disease. All the humours and coats were more or less destroyed ; the choroid, as far as it could be recognized, was converted into a dark, compact, pigmentous substance ; and the excised portion of the optic nerve was much altered in appearance, and surrounded by large fungous tubercles of brain-like consistence, which projected towards the circumference of the orbit, and "appeared to have been the matrix of the disease."

So much for fungus hæmatodes of the eye, a horrible and most malignant disease. The chances of success from an operation are faint enough, Heaven knows, when performed in the early stage of the malady, but the consequences of extirpation when ulceration has once taken place are well embodied in the details of the foregoing case.

CASE 2. *Fungus Hæmatodes of the Submaxillary Gland.*

Mrs. S. æt. 38, seven months gone with child, applied as an out-patient with a swelling beneath the skin about the size of a large walnut, of a conical figure, quite immoveable, and apparently firmly attached to the symphysis of the jaw—over the most prominent part the skin was of dull red colour, and it gave the sensation of fluctuation. She was emaciated and weak, and complained that her health had been deranged for some months. The tumour had scarcely existed two months. The patient considered it an abscess and was anxious that it should be opened but this "of course" was not complied with, and merely a belladonna plaster applied. The patient did not return for upwards of three weeks, when the tumour was the size of a very large orange, and from its centre issued a fungus reaching as low as the sternum, and bleeding freely at the slightest touch. It appeared that on the day she left the hospital she had induced a surgeon to puncture it with a lancet. Three or four ounces of blood escaped from the opening, the wound would not close, and in less than eight hours the fungus made its appearance, and subsequently continued rapidly to increase.

An opiate poultice and opium in pill were prescribed, but at the expiration of five days the patient was suddenly taken in labour, gave birth to a healthy boy, and died in twelve hours after her accouchement. No examination of the body was permitted.

The reporter remarks that most probably some important visceral disease existed, or death would not have ensued so prematurely. This is a conjecture which may or may not have been the fact; for our own parts we doubt it. The reporter likewise suspects that the disease originated from the periosteum of the maxilla, another conjecture which has not been put *hors de combat* by dissection. Of one thing we are very sure from the results of some experience, viz. that fungus hæmatodes does not originate so frequently in the periosteum as is commonly believed. Had we time and space we could mention some interesting cases in confirmation of our opinion, but we prefer saying a word or two on a practical point, the puncture of the tumour. Every one knows that fungus hæmatodes often presents a deceptive appearance of fluctuation, and that good surgeons have punctured it for abscess. But every one is not aware of the occasional bad effects of such an error. In the present instance a fungus arose from the wound in the short space of eight hours, and in another case which fell under our own observation the result was fatal. The case to which we refer occurred in a public hospital ; it was one of a tumour on the head which looked like an encysted tumour and had been punctured by a country surgeon. A probe was introduced and a good deal of exploration instituted, strange constitutional symptoms, with delirium, succeeded, and in two or three days the patient died. On dissection the disease was found to have been malignant fungus closely allied to the hæmatoid disease of Hey, springing from the cranial diploë, and destroying both inner and outer table of the skull. We think these cases, and they are by no means solitary, ought to teach practitioners caution in thrusting their fingers and their lancets into every tumour that falls in their way.

CASE 3. *Fungus Hæmatodes of the Breast.*

A. S. æt. 24, unmarried servant, admitted with a formidable enlargement of the left breast, which presents a capacious ulcerated opening at its upper part and a sloughy fungus projecting through it—breast elastic and very heavy—integuments uniformly smooth, but thickly studded with enlarged veins—axillary glands enlarged—no pain. Her health is completely broken up—constant short cough without expectoration—shortness of breath—great weakness without emaciation—expression of countenance anxious and left cheek constantly flushed—loss of appetite—little sleep—pulse 130, and small—catamenia regular.

Six months ago received an accidental injury on the part, and in a few days the whole mamma became extremely tender and sore when pressed, with much general swelling, but no redness. Leeches and lotions were repeatedly applied without benefit, for the breast daily increased in size and deep-seated pain supervened, which extended into the axilla and beneath the scapula. Six weeks ago had a severe attack of erysipelas over the whole breast, side, and back, which lasted six days, and terminated in the ulceration beforementioned through which the fungus has protruded. With the disappearance of the erysipelas she lost all pain.

Under these melancholy circumstances the operation was very properly considered inadmissible, and the patient returned to her friends, with whom after lingering for three weeks she died. Her mother and three other members of the family had died of the same disease.

Some ignorant humanity-mongers might think perhaps that the surgeon, Mr. Lyford, did wrong in discountenancing an operation in the foregoing case. We will tell them the results in an instance of a similar kind. A woman was admitted into St. George's Hospital with ulcerated fungus hæmatodes of the breast. The disease was extirpated by Mr. Keate, the wound rapidly cicatrized, and the patient left the hospital. In a month or thereabouts she returned with several tumours on the head. Paralysis and coma supervened, and the patient died a miserable object. On dissection there were four or five tumours of fungus hæmatodes growing from the cranial diploë, pushing inwards on the dura mater, and outwards through the external table; one of them entered the orbit. The lungs presented a few medullary tubercles; the liver was almost destroyed by them. This case shews the strong tendency to the recurrence of the disease after removal of the ulcerated fungus, but patients not unfrequently die of the immediate effects of the operation. A middle aged woman was lately operated on at St. George's Hospital for an ulcerated tumour of the breast, something between scirrhus and fungus hæmatodes. In a day or two erysipelas appeared, attended with nervous symptoms, and low constitutional disturbance. The patient became delirious, or rather maniacal, for there was no tendency to violence; the erysipelas faded, and at the end of about five or six days from the operation she died. On dissection there was nothing found to account for the fatal result. We mention these cases to illustrate the dangers and gloomy issue of operations for ulcerated fungus. We are not amongst those who think much of the powers of surgery even in the earlier stage of the disease, but nevertheless we are bound to say that the prospect is brighter under such circumstances. A young woman had the thigh removed near the hip-joint for fungus hæmatodes, not ulcerated, and although it is upwards of a year since the operation we believe there has been no return of the disease. Many persons would esteem even the chance of such fresh lease of life as this worth the pain, and misery, and risks of an operation. They should have their option, and it is some satisfaction to be able to assure them that they *may*, under favourable circumstances, rub on without a return of the disease, for several years.

